# Probing the Activity Enhancement of Carbocatalyst with the Anchoring of Atomic Metal

**DOI:** 10.3390/nano13172434

**Published:** 2023-08-27

**Authors:** Zhe Zhang, Jie Huang, Wei Chen, Jufang Hao, Jiangbo Xi, Jian Xiao, Baojiang He, Jun Chen

**Affiliations:** 1 School of Chemistry and Environmental Engineering, Key Laboratory of Novel Biomass-Based Environmental and Energy Materials in Petroleum and Chemical Industry, Key Laboratory of Green Chemical Engineering Process of Ministry of Education, Engineering Research Center of Phosphorus Resources Development and Utilization of Ministry of Education, Hubei Key Laboratory of Novel Reactor and Green Chemical Technology, Wuhan Institute of Technology, Wuhan 430205, China; z1094439949@163.com (Z.Z.); hjwitkl@163.com (J.H.); wchen@wit.edu.cn (W.C.); 2 Staff Development Institute of China National Tobacco Corporation (CNTC), Zhengzhou 450008, China; haojufang1982@126.com; 3 School of Chemical Engineering and Pharmacy, Wuhan Institute of Technology, Wuhan 430205, China; 4 Zhengzhou Tobacco Research Institute of China National Tobacco Corporation (CNTC), Zhengzhou 450001, China

**Keywords:** N, S co-doped holey graphene, atomic Pd, activity enhancement, nitroarene reduction

## Abstract

Enhanced catalysis for organic transformation is essential for the synthesis of high-value compounds. Atomic metal species recently emerged as highly effective catalysts for organic reactions with high activity and metal utilization. However, developing efficient atomic catalysts is always an attractive and challenging topic in the modern chemical industry. In this work, we report the preparation and activity enhancement of nitrogen- and sulfur-codoped holey graphene (NSHG) with the anchoring of atomic metal Pd. When employed as the catalyst for nitroarenes reduction reactions, the resultant Pd/NSHG composite exhibits remarkably high catalytic activity due to the co-existence of dual-active components (i.e., catalytically active NSHG support and homogeneous dispersion of atomic metal Pd). In the catalytic 4-nitrophenol (4-NP) reduction reaction, the efficiency (turnover frequency) is 3.99 × 10^−2^ mmol 4-NP/(mg cat.·min), which is better than that of metal-free nitrogen-doped holey graphene (NHG) (2.3 × 10^−3^ mmol 4-NP/(mg cat.·min)) and NSHG carbocatalyst (3.8 × 10^−3^ mmol 4-NP/(mg cat.·min)), the conventional Pd/C and other reported metal-based catalysts. This work provides a rational design strategy for the atomic metal catalysts loaded on active doped graphene support. The resultant Pd/NSHG dual-active component catalyst (DACC) is also anticipated to bring great application potentials for a broad range of organic fields, such as organic synthesis, environment treatment, energy storage and conversion.

## 1. Introduction

Heteroatom-doped carbonaceous materials (N [1,2], P [3], S [4] etc.) have drawn tremendous attention in the past few decades due to their unique physical and chemical properties. Many works have demonstrated that introducing different foreign atoms into the carbon matrix could change the charge density and spin density distributions of the neighboring carbon (α-C) atoms, which can serve as catalytically active sites for varieties of reactions [5,6]. Co-doping with different foreign atoms (e.g., two kinds of heteroatoms) could further regulate the geometric and electronic structures of α-C atoms, endowing the co-doped carbon materials with enhanced catalytic capability compared with their mono-heteroatom doped counterparts [7,8,9]. The two kinds of co-doped heteroatoms could modify the polarity of the carbon matrixes due to their different electronegative properties. For instance, the α-C atoms in N doped graphene possess substantially higher positive charge density compared with the C atoms in pristine graphene. After introducing additional S dopants, the charge balance of the graphene’s electron system could be broken, leading to a disrupted charge redistribution [10]. As a result, the dual-doping of S and N may introduce asymmetrical spin and charge density in NSHG and thus improve the electron transport. Benefitting from these unique properties, dual heteroatom co-doping carbon materials exhibit a broader range of applications [11,12,13]. As a result, these materials have been demonstrated as potential metal-free carbocatalysts in a variety of reactions, such as organic transformations [14] and electrochemical reactions [15]. Generally, heteroatoms are prone to be incorporated into the carbon networks at defected sites (i.e., edge, hole or vacancy) [16,17], which feature high accessibility for the active sites. In this aspect, holey graphene with a high density of mesopores possesses distinct structural defects, which can increase the heteroatom doping levels in the carbon frameworks [18]. Therefore, the high dopants concentration and specific surface area (SSA) make holey graphene to be an ideal candidate support material for the preparation of a high-performance metal-based catalyst [19]. However, 2D graphene nanosheets and derived materials are inclined to irreversibly restack due to the van der Waals forces (i.e., π-π stacking) [20], which would result in a decreased surface area and the exposure of reactive regions. In addition, the restacked graphene sheets may further block their partial mass transfer channels or thereby shield the active species in the corresponding anchoring sites, leading to a deterioration of the catalytic activity. To preserve the intrinsic properties of isolated graphene nanosheets, an effective way is to assemble graphene sheets into 3D architectures with interconnected networks and randomly dispersed pores. In recent years, many sophisticated strategies for the construction of 3D graphene architectures have been developed [21]. The gelation of graphene oxide (GO) [22], the hydrothermal assembling of GO [23], and the freeze drying and reduction of GO are effective and commonly used approaches that paved a way towards high-performance graphene-based materials for practical applications [24,25]. The assembled skeleton can not only enhance the accessibility of reactive regions or active species but also facilitate the mass transport and diffusion of reactants during the reaction process, thereby guaranteeing a high catalytic activity.

Besides creating active sites, the heteroatoms doped in graphene possess favorable coordination ability for metal species [26,27]. Heteroatom-doped graphene can be used as a legating material for metal anchoring via a strong metal-support interaction, which may optimize the electronic structure of metal, thus leading to a satisfied catalytic performance [28]. In this context, various heteroatom-co-doped graphene with high SSA have been used as supporting materials for the anchoring of nanosized or even atomic metal species. For instance, Li and coworkers supported Pd nanoparticles (NPs) on N, S co-doped carbon sphere via the coordinated effect of N/S dopants, which contributes to the formation of electron-rich defect surfaces and thus facilitates the stabilization of Pd NPs [29]. Tremendous efforts have been devoted to facilitating the metal dispersing, increasing the number of metal sites, and improving the utilization efficiency of the metal (especially noble metal) components. Liu’s coworkers supported Pd single-atoms on ordered porous N,S-doped carbon and achieved a highly efficient, stable alkaline hydrogen oxidation reaction [30]. Recent studies have demonstrated that the co-existence of metal atoms and metal NPs enhanced the activity of atomic catalysts due to the electronic interactions between them [31,32,33,34,35,36]. However, up to now, the incorporation of well-dispersed atomic and nanoscaled metal species onto the dual- or multi-heteroatom-co-doped porous graphene architecture still remains a great challenge in the catalysis field.

Inspired by the synergistic effects of metal with different sizes as well as heteroatom-co-doped graphene, we propose a combination strategy for the fabrication of dual-active component catalyst (DACC) with Pd species (i.e., Pd atoms and NPs) supported on catalytically active nitrogen- and sulfur-codoped holey graphene (NSHG) (Pd/NSHG). Owing to the combinational effect of the active NSHG carbocatalyst and Pd species, the resultant Pd/NSHG DACC shows enhanced catalytic activity toward nitroarenes reduction reactions.

## 2. Materials and Methods

### 2.1. Materials

Hydrogen peroxide (H_2_O_2_), nitrobenzene, 4-nitroanisole, 4-nitrotoluene, 4-nitroaniline, 4-chloronitrobenzene, 4-bromonitrobenzene, 4-nitrobenzoic acid, 4-nitrobenzonitrile, 3-bromonitrobenzene, 3-chloronitrobenzene, 3-nitrobenzoic acid, 4-nitrostyrene, 4-nitrophenol (4-NP), thiourea, urea, and sodium borohydride (NaBH_4_) were purchased from Sinopharm Chemical Reagent Co, Ltd. (Shanghai, China). Potassium tetrachloropalladate (K_2_PdCl_4_) was procured from Aladdin Chemistry Co., Ltd. (Shanghai, China). The rest of the chemicals were obtained from Sigma-Aldrich. Deionized water was used for all synthesis and experiments.

### 2.2. Preparation of Holey Reduced Graphene Oxide (HRGO)

GO aqueous dispersion was firstly prepared by a modified Hummers’ method [37]. The concentration of GO dispersion was determined via a freeze-drying method before use. 7 mL of H_2_O_2_ (0.3 wt.%) was injected into 60 mL of GO aqueous suspension (4.0 wt.%) under stirring. The obtained H_2_O_2_-GO aqueous suspension was transferred into a Teflon-lined autoclave (100 mL) and heated at 180 °C. After an 8 h hydrothermal treatment, GO nanosheets were etched, reduced and self-assembled to form a 3D architectural HRGO hydrogel. Finally, the obtained HRGO hydrogel was rinsed with deionized water.

### 2.3. Preparation of NSHG and Nitrogen-Doped Holey Graphene (NHG)

The as-prepared 3D HRGO hydrogel was firstly smashed and dispersed in 30 mL deionized water under sonication treatment. The uniform HRGO suspension was then freeze-dried to obtain 109.5 mg black powder. The powdery HRGO was subsequently mixed with 3.3 g (43.35 mmol) solid thiourea to form a uniform HRGO-thiourea composite. Furthermore, the HRGO-thiourea composite was heated to 700 °C within 60 min and maintained for an extra 2 h under a 50 sccm of N_2_ flow. In the annealing process, thiourea decomposed and subsequently released gaseous NH_3_ and H_2_S and CS_2_, which can result to N and S co-doping in the graphene framework [38]. Finally, the sample was cooled to room temperature under ambient N_2_ and collected to obtain 97.8 mg of NSHG. For comparison, NHG was prepared similarly to that of NSHG except substituting thiourea with urea.

### 2.4. Preparation of Pd/NSHG DACC

The resultant NSHG (87.0 mg) was dispersed in ca. 50 mL water by pulverization treatment for 3 h, and a homogeneous NSHG suspension can be obtained. Then, 10 mL aqueous K_2_PdCl_4_ (5.4 mg, 0.016 mmol) solution was added to the suspension and stirred for 3 h in an ice bath. During this process, Pd atoms, clusters and NPs were deposited on the support by a facile and green method via redox reaction between PdCl_4_^2−^ and adsorption of PdCl_4_^2−^ by NSHG, leading to the formation of the Pd/NSHG DACC. Afterwards, the reaction mixture was filtered and washed three times with pure water to remove the remaining reagents.

### 2.5. Activity Evaluation and Kinetic Study of Pd/NSHG DACC for 4-NP Reduction Reaction

The reaction was carried out in a 50-mL glass vial at room temperature (ca. 25 °C) and atmospheric pressure. In a typical reaction run, 0.3 mg Pd/NSHG DACC, 4-NP aqueous solution (10.0 mL, 20.0 mM, 0.2 mmol) and NaBH_4_ (756.0 mg, 20.0 mmol) were mixed in the vial under stirring. The reaction process was constantly monitored by observation of color changes and UV-Vis analysis. For kinetic study of Pd/NSHG DACC catalyzed 4-NP reduction reaction, 0.3 mL of reaction mixture was withdrawn and filtered to remove the catalyst. After that, the reaction mixture was diluted to 0.1 mM and subsequently analyzed by UV-Vis absorption spectrometry.

### 2.6. Durability Test of Pd/NSHG DACC

The durability and stability tests of Pd/NSHG DACC was carried out in a 50-mL glass vial at room temperature (ca. 25 °C) and atmospheric pressure. Pd/NSHG DACC (3.0 mg) was added to the mixture of 4-NP (10.0 mL, 20 mM, 0.2 mmol) and NaBH_4_ (756.0 mg, 2.0 mol) aqueous solution under vigorous stirring. After each catalytic run, the catalyst was collected from the reaction mixture by filtration and washed with deionized water. The recovered catalyst was reused for the next run to repeat the 4-NP reduction reaction.

### 2.7. Activity Evaluation of Pd/NSHG DACC for the Reduction of Other Nitroarenes

The reaction was carried out in a 5-mL glass vial at room temperature (ca. 25 °C) under atmospheric pressure. In a typical procedure, the Pd/NSHG (2.0 mg) suspension was added to 3 mL of aqueous ethanol solution (H_2_O/ethanol = 1/9 *v*/*v*) solution together with other nitroarene substrate (0.06 mmol) and NaBH_4_ (6 mmol). Then the reaction mixture was thoroughly mixed with magnetic stirring under ambient conditions. The reaction process was constantly monitored by thin-layer chromatography (TLC) at regular intervals. After completion of the reactions, the reaction mixture was filtrated to remove the catalyst and subsequently analyzed by high-performance liquid chromatography (HPLC).

## 3. Results and Discussion

### 3.1. Preparation and Caracterization of Pd/NSHG DACC

**Figure 1:**
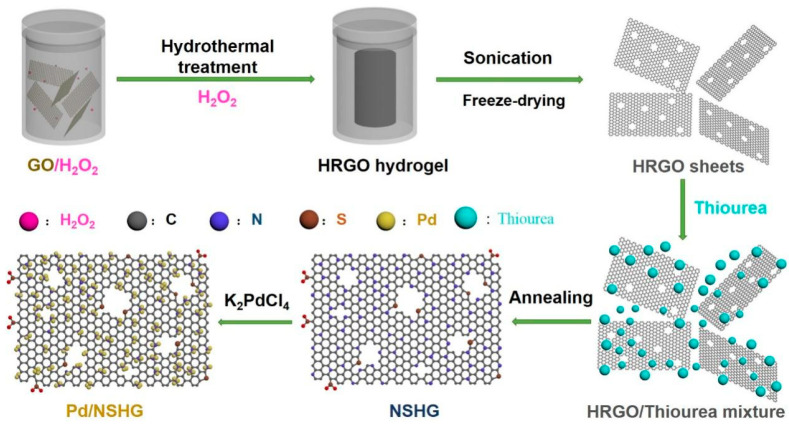
Schematic illustrating the fabrication process of Pd/NSHG DACC.

The Pd/NSHG DACC was synthesized by four steps as shown in Figure 1. Firstly, GO nanosheets were etched, reduced and self-assembled to form a cylinder-shaped HRGO hydrogel through a hydrothermal treatment (Figure S1a). The obtained 3D architectural HRGO hydrogel was then smashed through sonication to get aqueous suspension (Figure S1b) and subsequently freeze-dried to afford powdery HRGO. After that, the resultant HRGO powder was mixed with thiourea and subsequently annealed to co-dope N and S atoms into the HRGO, leading to the formation of NSHG. Finally, atomic and nanosized Pd species were introduced onto NSHG to prepare Pd/NSHG DACC by a facile immersing method.

**Figure 2:**
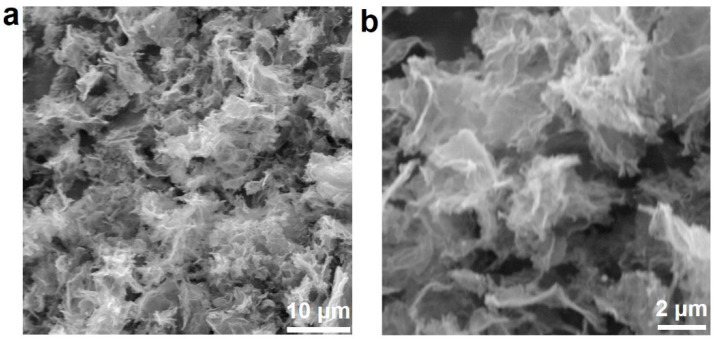
(**a**,**b**) SEM images Pd/NSHG DACC with different magnifications.

**Figure 3:**
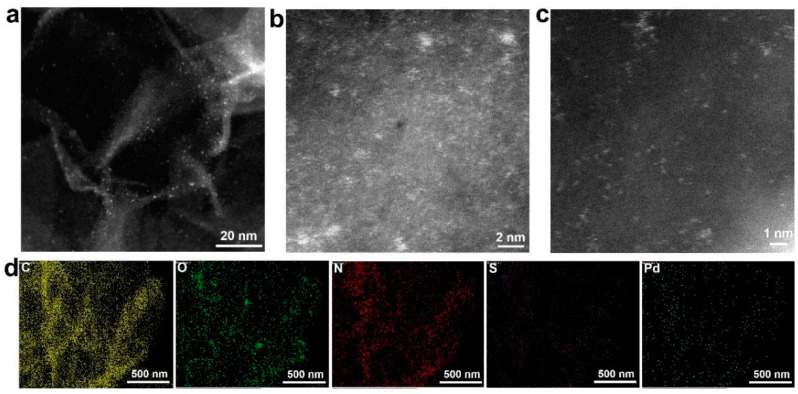
(**a**) HAADF-STEM and (**b**,**c**) high-resolution HAADF-STEM images of Pd/NSHG DACC, (**d**) C, O, N, S, and Pd elemental mapping of Pd/NSHG DACC.

The morphology and microstructure of the as-prepared Pd/NSHG DACC were carefully examined by means of scanning electron microscopy (SEM) and transmission electron microscopy (TEM). As displayed in Figure 2, the typical SEM images of Pd/NSHG DACC revealed an interconnected 3D architecture constructed by randomly aggregated graphene nanosheets. Similar interconnected architecture of graphene sheets was also observed in NHG sample (Figure S2). Aberration-corrected high-angle annular dark-field (HAADF) scanning transmission electron microscopy (STEM) images showed that Pd/NSHG DACC possessed a typical curved sheet-like structure. In addition, randomly dispersed pores (black holes) and metal species (bright dots) could be clearly observed from HAADF-STEM images (Figure 3a–c), confirming the successful etching of graphene sheets and the loading of Pd species on NSHG support. According to the sub-nanometer sizes, the Pd species were mainly existed as atomic metals, such as single-atoms and clusters. The energy dispersive spectroscopy (EDS) elemental mapping analysis revealed that the C, O, N, S, and Pd elements were homogeneous distributed in the Pd/NSHG DACC sample, further confirming the co-doping of N and S and the incorporation of Pd in NSHG (Figure 3d).

**Figure 4:**
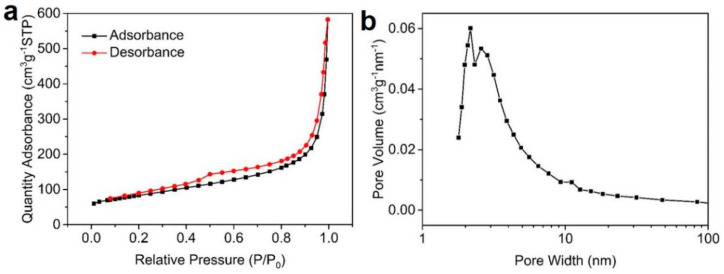
(**a**) Nitrogen adsorption/desorption isotherms of Pd/NSHG DACC and (**b**) pore-size distributions derived from desorption branch of the isotherms.

N_2_ adsorption/desorption isotherm of Pd/NSHG DACC exhibited a type IV N_2_ adsorption-desorption hysteresis loop at a relative pressure between ca. 0.5 and 0.9, illustrating the co-existence of mesopores and macropores in NSHG support. The specific surface area of Pd/NSHG DACC was as high as 291.8 m^2^/g calculated by Brunauer-Emmett-Teller method (Figure 4a), indicating that not serious aggregation was caused by π-π stacking of NSHG nanosheets when annealed with thiourea. The pore-size analysis revealed that Pd/NSHG DACC had a multiple pore structure with a distribution from 2 to 10 nm (Figure 4b); These mesopores could facilitate the diffusion efficiency of reactants and enhance the exposure of active sites including both the activated C sites and Pd sites during the catalytic process.

**Figure 5:**
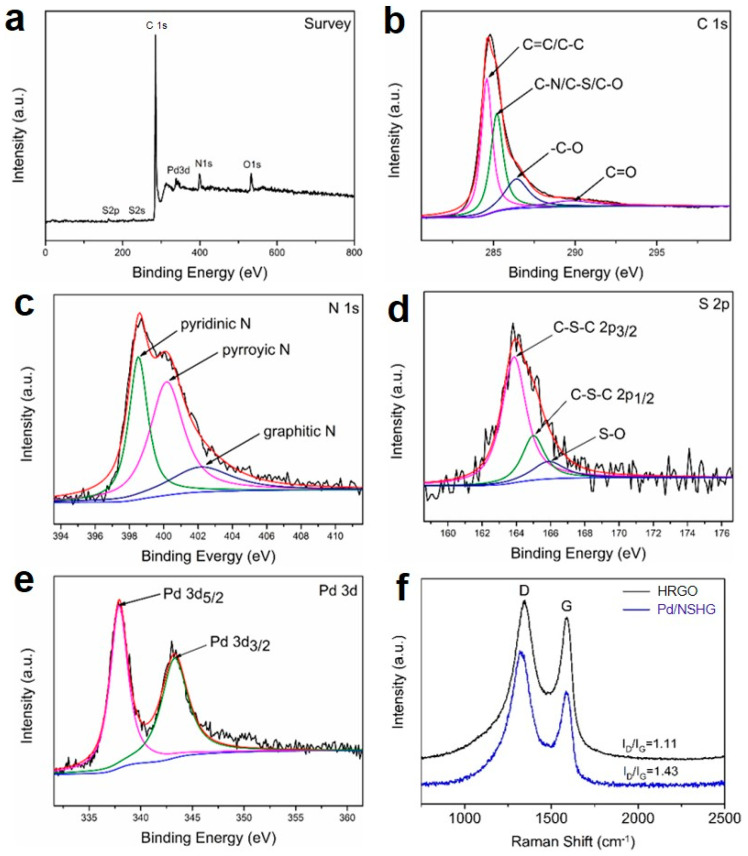
(**a**) XPS survey spectrum of Pd/NSHG DACC, high resolution spectra of (**b**) C 1s, (**c**) N 1s, (**d**) S 2p, (**e**) Pd 3d for Pd/NSHG DACC, (**f**) Raman spectra of HRGO (black line) and Pd/NSHG DACC (blue line).

Moreover, X-ray photoelectron spectroscopy (XPS) was used to get insight of the surface composition information, including the atomic content and bonding states of each element in Pd/NSHG DACC. As can be seen from the XPS survey spectrum of Pd/NSHG DACC (Figure 5a), there were six characteristic peaks at the binding energy of ca. 164, 228, 285, 340, 399, and 533 eV, corresponding to S 2p, S 2s, C 1s, Pd 3d, N 1s, and O 1s, respectively [38,39]. The atomic content of N and S elements in NSHG were 6.72% and 1.18%, respectively, indicating the desired co-doping of N and S in graphene. These incorporated N and S dopants could create metal-free active C sites by interrupting the graphitic hexagonal carbon configuration and regulating the electronic structure of graphene framework [10,11]. The relatively low S content should be attributed to the larger atom radius (1.03 Å vs. 0.71 Å of N and 0.75 Å of C), which made S atoms more difficult to incorporate into the graphitic structure [11]. In addition, the co-doped N and S atoms may facilitate the anchoring of metal on NSHG via heteroatom-metal coordination due to the strong support-metal interactions. In the deconvoluted C 1s spectrum, the peaks located at ca. 284.6 eV, 285.2 eV, 286.4 eV, and 290.0 eV could be assigned to C=C or C-C bond, C-O, C-N or C-S bond, C=O bond, and O-C=O bond, respectively (Figure 5b) [11]. The high-resolution N 1s spectrum could be fitted into three peaks, corresponding to pyridinic N (398.6 eV), pyrrolic N (400.2 eV) and graphitic N (402.2 eV) species (Figure 5c) [40]. The S 2p spectrum could be fitted into three components (Figure 5d), corresponding to S 2p3/2 (163.9 eV) and S 2p1/2 (165.0 eV) of thiophene-like configuration, and sulfur oxide (SO_x_) (165.9 eV) [41,42]. Moreover, the Pd 3p signal revealed the presence of Pd 2p3/2 (337.6 eV) and Pd 2p1/2 (343.0 eV) of Pd (II) species (Figure 5e) [43]. For precisely quantitative measurement of metal loading, inductively coupled plasma mass spectroscopy (ICP-MS) was used to determine the of the Pd content, which was 1.34 wt.%. Raman spectroscopy measurements demonstrated that the intensity ratio of the D band at ca. 1350 cm^−1^ and G band at ca. 1580 cm^−1^ (I_D_/I_G_) of Pd/NSHG DACC (1.43) was much higher than that of HRGO (1.11). The finding indicated that NSHG possessed a higher defect level than HRGO due to the co-doping of heteroatoms (i.e., N and S) which could break the structural symmetry of carbon in graphene framework [44].

### 3.2. Catalytic Property of NHG Carbocatalyst, NSHG Carbocatalyst and Pd/NSHG DACC

**Figure 6:**
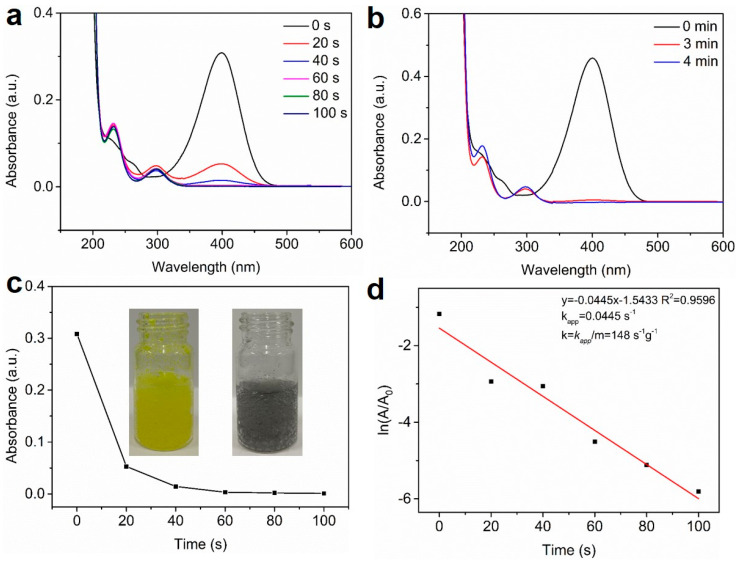
(**a**) UV-vis absorption spectra of the reaction solution of 4-NP reduction catalyzed by Pd/NSHG DACC and (**b**) metal-free NSHG carbocatalyst. The plot of (**c**) A/A_0_ and (**d**) ln(A/A_0_) of 4-NP-NaBH_4_ aqueous solution against time catalyzed by the Pd/NSHG DACC. The inset in (**c**) showing a photograph of the reduction process of 4-NP-NaBH_4_ aqueous solution in the presence of Pd/NSHG DACC.

**Figure 7:**
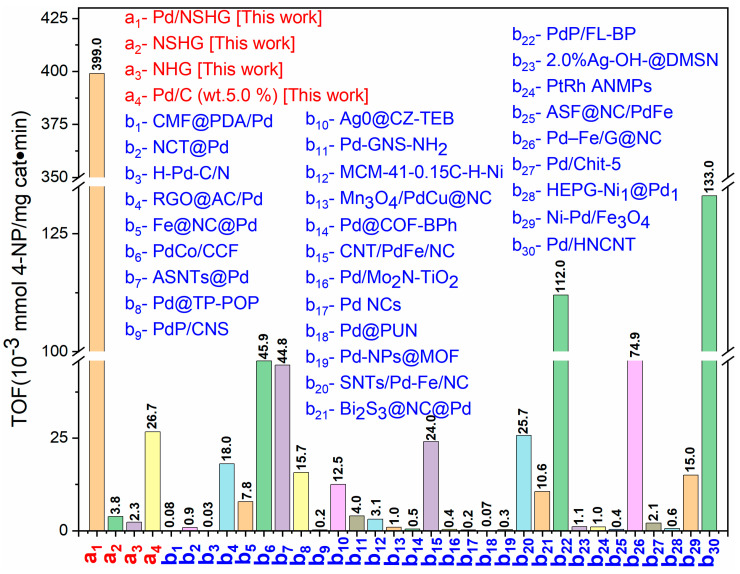
The catalytic efficiency (TOF) towards 4-NP reduction of Pd/NSHG DACC, NHG and NSHG carbocatalysts, and other recently reported noble metal-based catalysts. b_1_–b_30_: data adapted from references [45,46,47,48,49,50,51,52,53,54,55,56,57,58,59,60,61,62,63,64,65,66,67,68,69,70,71,72,73,74].

Firstly, the reduction 4-NP to 4-aminophenol (4-AP) was chosen as a representative model reaction to evaluate the catalytic performance of metal-free NHG and NSHG carbocatalysts as well as Pd/NSHG DACC toward the reduction of nitroarenes. We noted that Pd/NSHG DACC exhibited remarkably high catalytic efficiency for a 4-NP reduction reaction as both atomic Pd and NSHG support were catalytically active in the reaction system. For an accurate quantitative evaluation of the catalytic performance, turnover frequency (TOF, defined here as the amount of 4-NP that 1 mg Pd/NSHG DACC can convert into 4-AP per min) (refer to the calculation formula) was employed to calculate the catalytic activity of the catalyst [16]. 0.2 mmol of 4-NP could be completely reduced into 4-AP by NaBH_4_ within 100 s in the presence of 0.3 mg of Pd/NSHG DACC (Figure 6a), showing a TOF value of 0.399 mmol/(mg cat.· min). For metal-free carbocatalysis, 0.06 mmol of 4-NP could be completely reduced into 4-AP within 240 s by NaBH_4_ with 4.0 mg of NSHG carbocatalyst (Figure 6b), showing a superior TOF value of 0.0038 mmol/(mg cat.· min) to NHG carbocatalyst (0.0023 mmol/(mg cat.· min), Figure S3). Due to the contribution of the two active components (i.e., atomic Pd species and active NSHG carbocatalyst), the Pd/NSHG DACC exhibited much higher catalytic efficiency for 4-NP reduction than that of metal-free NHG and NSHG carbocatalysts, commercial Pd/C (5.0 wt.%) benchmarks, and recently reported metal-based catalysts (Figure 7 and Table 1) [45,46,47,48,49,50,51,52,53,54,55,56,57,58,59,60,61,62,63,64,65,66,67,68,69,70,71,72,73,74]. Furthermore, kinetics study was also performed to quantitatively evaluate the catalytic activity of Pd/NSHG DACC. As displayed in Figure 6c, ln(A) (A is the absorbance intensity at 400 nm for aqueous 4-NP-NaBH_4_ mixed solution) was linearly correlated with reaction time, confirming the pseudo-first-order reaction process [70]. The apparent rate constant (k_app_) determined from the slope was 4.45 × 10^−2^ s^−1^ for 4-NP reduction (Figure 6d), which was calculated based on total weight of active components, including metal Pd and NSHG. The corresponding activity factor (k) of Pd/NSHG DACC was 148.0 s^−1^ g^−1^. In addition, the Pd/NSHG DACC maintain ca. 45% catalytic activity (TOF) of the freshly prepared catalyst after 7 cycles, displaying favorable durability and stability (Figure S4). Since the substrate tolerance is another significant criterion to evaluate the application potential of catalyst [75]. We further extended the scope to other nitroaromatic substrates. Findings showed that Pd/NSHG DACC displayed good tolerance to a broad spectrum of substituted nitroarenes, such as nitrobenzene, 4-nitroanisole, 4-nitrotoluene, 4-nitroaniline, 4-chloronitrobenzene, 4-bromonitrobenzene, 4-nitrobenzoic acid, 4-nitrobenzonitrile, 3-bromonitrobenzene, 3-chloronitrobenzene, 3-nitrobenzoic acid, and 4-nitrostyrene. The catalytic reduction of these nitroaromatic substrates could also be achieved with high yields (Table 2), indicating the high catalytic activity and excellent generality of Pd/NSHG DACC. It should be noted that the selectivity was over 85%, even though there were reducible substituted groups (e.g., –COOH, –C=C, and –C≡N functional groups) in the molecular structures.

$$\mathrm{TOF}\left(\min^{-1}\right)=\frac{\mathrm{mmoles}\;\mathrm{of}\;4-\mathrm{NP}\;\mathrm{converted}\;\mathrm{per}\;\mathrm{minute}}{\mathrm{mg}\;\mathrm{of}\;\mathrm{Pd}/\mathrm{NSHG}\;\mathrm{catalyst}}$$

## 4. Conclusions

In summary, the synthesis of Pd/NSHG DACC and the activity enhancement of the NSHG carbocatalyst by the decoration of well-dispersive atomic Pd have been achieved. Benefiting from its unique structural merits, such as a large specific surface area, the existence of catalytically active NSHG support, as well as the homogeneous dispersion of atomic Pd species, the resulting Pd/NSHG DACC was endowed with remarkably high catalytic activity and selectivity toward nitroarenes reduction reactions. In the catalytic 4-NP reaction, its catalytic performance was better than that of metal-free NHG and NSHG carbocatalysts, commercial Pd/C, and conventional metal-based catalysts. This work offers an effective approach to the fabrication of catalysts with the highly efficient atomic Pd species loaded on heteroatom-doped holey graphene supports. Furthermore, the insights from the integration of active metal species and metal-free carbocatalyst into a DACC is anticipated to propose a sophisticated strategy for the activity enhancement in a synergistic manner. The DACCs are also expected to show great application potentials in a broad range of fields, including organic synthesis, environment treatment, energy storage and conversion, etc.

## Figures and Tables

**Table 1:** The comparison of catalytic activity of Pd/NSHG, NSHG and NHG carbocatalysts, commercial Pd/C (5.0 wt.%) and other metal-based catalysts for 4-NP reduction.

Catalysts	Mass of Catalyst (mg)	Amount of 4-NP (mmol)	Conversion Time (min)	TOF (mmol 4-NP/(mg cat.·min)	Ref.
Pd/NSHG	0.3	0.2	1.67	3.99 × 10^−1^	a
NSHG	4.0	0.06	4.0	3.8 ×10^−3^	a
NHG	4.0	0.06	8.67	2.3 ×10^−3^	a
Pd/C (5.0 wt.%)	1.0	0.2	7.5	2.67 × 10^−2^	a
CMF@PDA/Pd	492.0	1 × 10^−2^	0.25	8.13 × 10^−5^	[45]
NCT@Pd	1.0	6 × 10^−4^	0.67	9 × 10^−4^	[46]
H-Pd-C/N	0.045	3 × 10^−4^	240	2.78 × 10^−5^	[47]
RGO@AC/Pd	1.0	3 × 10^−2^	1.65	1.8 × 10^−2^	[48]
Fe@NC@Pd	2.0	3 × 10^−2^	1.92	7.8 × 10^−3^	[49]
PdCo/CCF	0.15	2.7 × 10^−2^	3.92	4.59 × 10^−2^	[50]
ASNTs@Pd	2.0	6 × 10^−2^	0.67	4.48 × 10^−2^	[51]
Pd@TP-POP	0.3	2.7 × 10^−2^	5.75	1.57 × 10^−2^	[52]
PdP/CNS	6 × 10^−2^	2.7 × 10^−4^	23	1.96 × 10^−4^	[53]
Ag^0^@CZ-TEB	2.0	5 × 10^−2^	2	1.25 × 10^−2^	[54]
Pd-GNS-NH_2_	5.0	2 × 10^−2^	1	4 × 10^−3^	[55]
MCM-41-0.15C-H-Ni	28.0	0.7	8	3.125 × 10^−3^	[56]
Mn_3_O_4_/PdCu@NC	4 × 10^−2^	2.7 × 10^−4^	7	9.64 × 10^−4^	[57]
Pd@COF-BPh	0.1	3 × 10^−4^	6	5 × 10^−4^	[58]
CNT/PdFe/NC	1.0	6 × 10^−2^	2.5	2.4 × 10^−2^	[59]
Pd/Mo_2_N-TiO_2_	10.0	6 × 10^−3^	1.67	3.6 × 10^−4^	[60]
Pd NCs	0.14	2 × 10^−4^	6	2.38 × 10^−4^	[61]
Pd@PUN	30.0	2 × 10^−3^	1	6.7 × 10^−5^	[62]
Pd-NPs@MOF	5.0	2.15 × 10^−3^	1.33	3.225 × 10^−4^	[63]
SNTs/Pd-Fe/NC	2.0	6 × 10^−2^	1.17	2.57 × 10^−2^	[64]
Bi_2_S_3_@NC@Pd	1.0	6 × 10^−2^	5.67	1.06 × 10^−2^	[65]
PdP/FL-BP	8.063 × 10^−3^	3 × 10^−4^	0.33	1.12 × 10^−1^	[66]
2.0%Ag-OH^−^@DMSN	0.15	5 × 10^−4^	3	1.11 × 10^−3^	[67]
PtRh ANMPs	2 × 10^−2^	4 × 10^−4^	20	1 × 10^−3^	[68]
ASF@NC/PdFe	32.0	3 × 10^−3^	0.27	3.5 × 10^−4^	[69]
Pd–Fe/G@NC	1.0	0.2	2.67	7.49 × 10^−2^	[70]
Pd/Chit-5	150	1	3	2.1 × 10^−3^	[71]
HEPG-Ni_1_@Pd_1_	0.2	2.5 × 10^−6^	2.03	6.1 × 10^−4^	[72]
Ni-Pd/Fe_3_O_4_	1	6 × 10^−2^	4	1.5 × 10^−2^	[73]
Pd/HNCNT	0.5	0.2	31.	1.33 × 10^−1^	[74]

a: This work.

**Table 2:** Reduction of nitroarenes into aminoarenes with NaBH_4_ catalyzed by Pd/NSHG DACC ^1^.

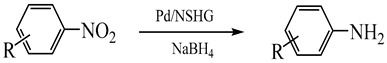					
Entry	Reactant	Product	Time (min)	Yield (%) ^2^	Selectivity (%) ^2^
1	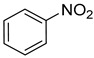	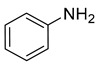	50.0	93.72	99.99
2	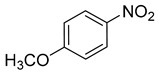	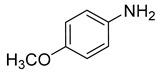	0.5	97.67	99.99
3	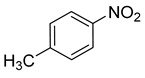	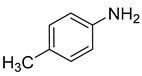	10.0	90.0	99.9
4	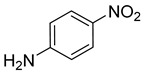	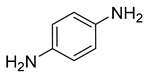	0.5	99.69	99.99
5	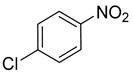	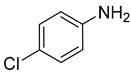	15.0	92.22	99.99
6	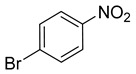	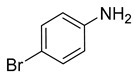	10.0	99.43	99.99
7	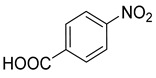	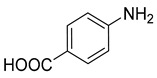	30.0	99.9	99.99
8	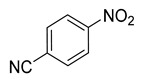	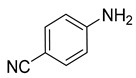	120.0	99.0	99.99
9	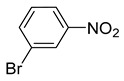	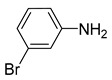	17.5	92.0	99.99
10	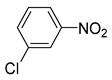	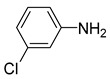	20.0	94.8	99.99
11	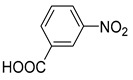	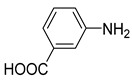	20.0	99.9	99.99
12	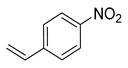	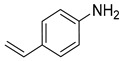	20.0	85.9	90.30

^1^ Reaction conditions: 0.06 mmol of nitroarenes, 2 mg of Pd/NSHG DACC, 3 mL of aqueous ethanol solution (H_2_O/ethanol = 1/9 *v*/*v*), 6 mmol of NaBH_4_, room temperature. ^2^ Determined by HPLC.

## Data Availability

Not applicable.

## Supplementary Materials

The following supporting information can be downloaded at: https://www.mdpi.com/article/10.3390/nano13172434/s1, Figure S1: The photograph of the 3D architectural HRGO hydrogel; Figure Figure S2: SEM images of NHG carbocatalyst; Figure S3: UV-Vis absorption spectra of the reaction solution of 4-NP reduction catalyzed by metal-free NHG catalyst; Figure S4: Catalytic efficiency (TOF) of Pd/NSHG catalyst for 4-NP reduction in each cycle.
