# Supplementary material for: Probing the Activity Enhancement of Carbocatalyst with the Anchoring of Atomic Metal
Source: Nanomaterials (Basel). 2023 Aug 27;13(17):2434. doi: 10.3390/nano13172434 (PMC10489856; doi:10.3390/nano13172434)
Supplement: Supplementary file 1 [file nanomaterials-13-02434-s001.zip › nanomaterials-2522972-supplementary.pdf]

Supplementary Materials

Probing the Activity Enhancement of Carbocatalyst with the Anchoring of Atomic Metal

Zhe Zhang [1], Jie Huang [1], Wei Chen [1], Jufang Hao [2], Jiangbo Xi [1,*], Jian Xiao [3,*], Baojiang He [4,*] and Jun Chen [1,*]

[1] School of Chemistry and Environmental Engineering, Key Laboratory of Novel Biomass-Based Environmental and Energy Materials in Petroleum and Chemical Industry, Key Laboratory of Green Chemical Engineering Process of Ministry of Education, Engineering Research Center of Phosphorus Resources Development and Utilization of Ministry of Education, Hubei Key Laboratory of Novel Reactor and Green Chemical Technology, Wuhan Institute of Technology, Wuhan 430205, China; z1094439949@163.com (Z.Z.); hjwitkl@163.com (J.H.); wchen@wit.edu.cn (W.C.)

[2] Staff Development Institute of China National Tobacco Corporation (CNTC), Zhengzhou 450008, China; haojufang1982@126.com

[3] School of Chemical Engineering and Pharmacy, Wuhan Institute of Technology, Wuhan 430205, China

[4] Zhengzhou Tobacco Research Institute of China National Tobacco Corporation (CNTC),

Zhengzhou 450001, China

* Correspondence: jbxi@wit.edu.cn (J.X.); jxiao@wit.edu.cn (J.X.); 15937100101@139.com (B.H.);

ychenjun2006@163.com (J.C.)

* Correspondence: jbxi@wit.edu.cn (J.X.); jxiao@wit.edu.cn (J.X.); ychenjun2006@163.com (J.C.) or 15937100101@139.com (B.H.)

Characterization techniques. The morphology and structure of products were characterized with a field-emission scanning electron microscope (SEM, Vega 3, TESCAN) and a transmission electron microscope (TEM, Tecnai G2 20, FEI). High-resolution transmission electron microscopy (HRTEM) was performed by employing FEI Titan 80–300 equipped with a spherical aberration (Cs) corrector for the objective lens. The HRTEM images were taken at a high tension of 300 kV, with the spherical aberration set at around -13 μm. Aberration-corrected high-angle annular dark-field scanning transmission electron microscopy (HAADF-STEM) images were acquired from a FEI Titan 80-300 microscope at 300 kV- HAADF-STEM images were acquired with the illumination semi-angle of 22 mrad and probe current of 50 pA. The dwell time for image acquisition was set at 10 μs per pixel to ensure a desirable signal-to-noise ratio. X-ray photoelectron spectroscopy (XPS) measurements were performed on VG ESCALAB 250 spectrometer with monochromatic Al Kα (1486.71 eV) X-ray radiation (15 kV and 10 mA) and hemispherical electron energy analyzer. The specific surface area was measured with Micromeritics ASAP2020. Surface area and porosity analysis were calculated using the Brunauer−Emmett−Teller (BET) equation. The Pd contents in the catalysts were determined using an inductivity coupled plasma-mass spectrometer (ICP-MS, Agilent 7700X, USA). The UV-Vis measurements were performed on a UV-2550 spectrophotometer (Shimadzu, Japan). High-performance liquid chromatography (HPLC) analysis was performed on an Agilent-1100 system with a Zorbax Eclipse XDB-C18 4.6×150 mm column (Agilent, USA).

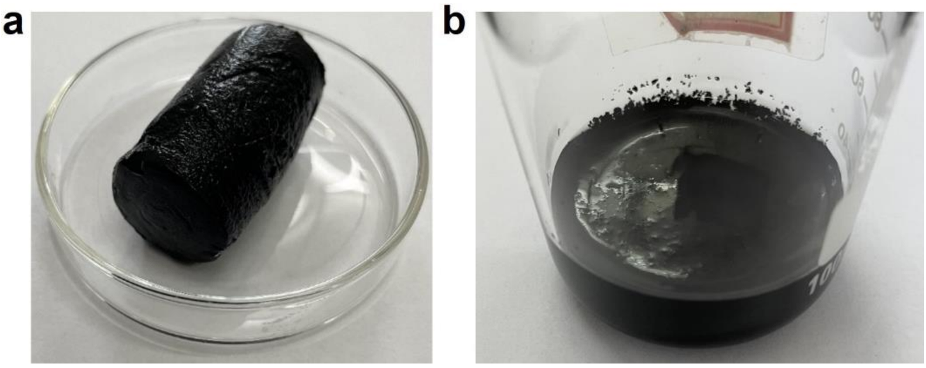

Figure S1 (a) The photograph of the 3D architectural HRGO hydrogel and (b) HRGO aqueous suspension.

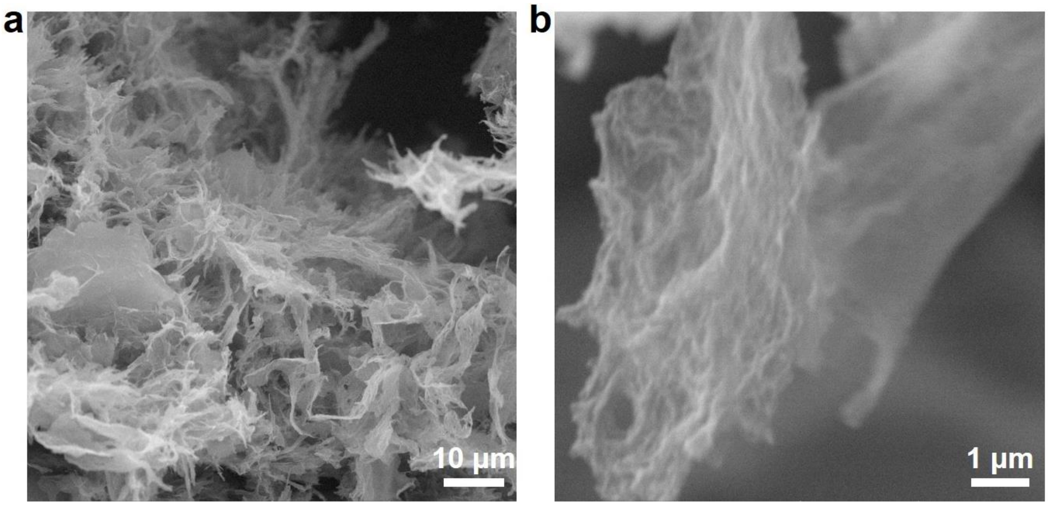

Figure S2. (a, b) SEM images of NHG carbocatalyst.

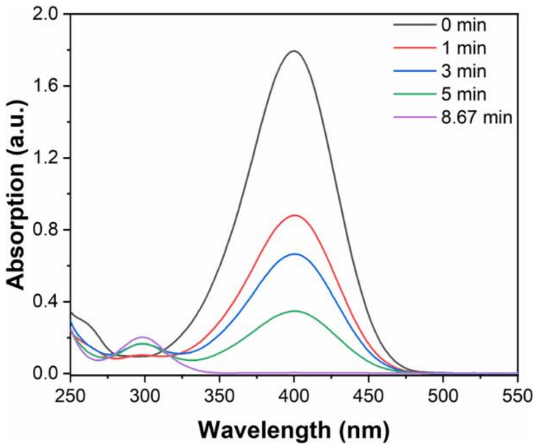

Figure S3 UV-Vis absorption spectra of the reaction solution of 4-NP reduction catalyzed by metal-free NHG carbocatalyst.

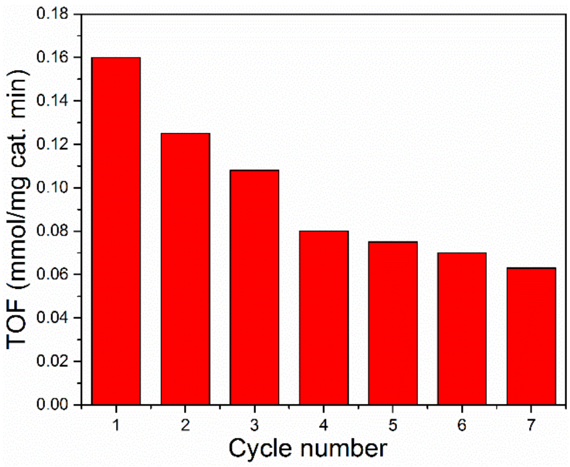

Figure S4 Catalytic efficiency (TOF) of Pd/NSHG DACC for 4-NP reduction in each cycle.
